# Stability of MR brain-perfusion measurement using arterial spin labeling

**DOI:** 10.1186/2197-7364-2-S1-A67

**Published:** 2015-05-18

**Authors:** Jan Petr, Frank Hofheinz, Ivan Platzek, Georg Schramm, Jorg Van Den Hoff

**Affiliations:** Helmholtz-Center Dresden-Rossendorf, PET Center, Institute of Radiopharmaceutical Cancer Research, Germany

Arterial spin labeling (ASL) is an MR technique for assessment of cerebral blood flow (CBF) that does not require use of contrast agents which makes it a less invasive alternative to the 15O-H2O-PET measurement. The repeatability of ASL has been studied extensively but mainly in young healthy volunteers. We have tested repeatability of ASL under realistic clinical conditions in elderly brain tumor patients acquired with a Philips Ingenuity TF PET/MR in the context of an ongoing 11C-Methionine PET/MR study. Twenty three patients (age 54.8±13.0 y) were scanned on two or more session. The patients underwent 6 weeks of concurrent radiochemotherapy with Temozolomide between the first session and second measurement. The mean relative difference of gray matter CBF was 18.6% between the first two session and 13.0% for the second session and further on. The mean gray matter CBF was 46.6±7.2 mL/min/100 g on the first sessions and there was a significant decrease of 9.8% between first and second session (p=0.027). In summary, the ASL presents measurement of CBF with reasonable repeatability also in elderly patients under clinical conditions when it is not possible to control for all sources of variation. Significant decrease of CBF in healthy tissue was observed after the radiochemotherapy. Prospectively, the ASL data together with the also acquired 11C-Methionine PET will be evaluated regarding their separate and combined ability to predict patient outcome and effectiveness of the performed radiochemotherapy.

